# Comparison of Two Manual Therapy Programs, including Tendon Gliding Exercises as a Common Adjunct, While Managing the Participants with Chronic Carpal Tunnel Syndrome

**DOI:** 10.1155/2022/1975803

**Published:** 2022-06-08

**Authors:** Fauzia Javed Sheereen, Bibhuti Sarkar, Pallavi Sahay, Mohammad Abu Shaphe, Ahmad H. Alghadir, Amir Iqbal, Taimul Ali, Fuzail Ahmad

**Affiliations:** ^1^Department of Cardiothoracic and Vascular Surgery, AIIMS, New Delhi, India; ^2^National Institute for Locomotor Disabilities (Divyangjan), Kolkata, India; ^3^Department of Physical Therapy, College of Applied Medical Sciences, Jazan University, Jazan, Saudi Arabia; ^4^Department of Rehabilitation Sciences, College of Applied Medical Sciences, King Saud University, P.O. Box 10219, Riyadh 11433, Saudi Arabia; ^5^Peerless Hospitex Hospital and Research Center, Kolkata, India; ^6^Department of Physical Therapy, College of Applied Medical Sciences, Majmaah University, Majmaah, Saudi Arabia

## Abstract

**Background:**

Carpal tunnel syndrome (CTS) is the symptomatic compression neuropathy of the median nerve at the wrist level that may become a reason for upper limb disability, in the women and men population.

**Objective:**

This study aimed to compare the efficacy of the neurodynamic technique (NT) and carpal bone mobilization technique (CBMT) incorporated with tendon gliding exercises (TGE) as an effect-enhancing adjunct while managing the participants with chronic CTS.

**Methods:**

The study followed a two-arm parallel-group randomized comparative design. Thirty participants (aged 30–59 years) with chronic CTS were recruited randomly to both the NT and CBMT groups. In addition to the TGE (a common adjunct), NT and CBMT were performed in the NT and CBMT groups, respectively, for three weeks. The primary outcome measures including pain intensity, functional status, grip strength, and motor nerve conduction study were assessed using a visual analogue scale (VAS), Boston Carpal Tunnel Questionnaire (BCTQ), hand-held dynamometer, and electromyograph, respectively, at baseline, 3 weeks postintervention, and follow-up at one week post end of the intervention. Paired and unpaired *t*-test were used to calculate the differences in intervention effects within and between the groups with keeping the level of significance *α* at 0.05.

**Results:**

The data analysis revealed a significant (95% CI, *p* < 0.05) difference for all outcomes within each group compared across different time intervals. Similarly, a significant difference was found for all outcomes except pain and grip strength compared between groups at 3 weeks postintervention and follow-up at one week post end of the intervention.

**Conclusions:**

The NT revealed more effectiveness than the CBMT when incorporated with TGE to improve nerve conduction velocity and functional status of the hand. However, both NT and CBMT were equally effective in improving pain and grip strength while managing the participants with chronic CTS. In addition, the TGE contributed as a beneficial, effect-enhancing adjunct to the NT and CBMT differently. *Significance*. The study will guide the physiotherapist in applying either of the combination techniques suitable for achieving treatment objectives while managing the participants with chronic CTS.

## 1. Introduction

Carpal tunnel syndrome (CTS) is a common median nerve compression syndrome at the wrist level (3.8%), most commonly affecting the female population and becoming a reason for upper limb disability [1, 2]. Acute carpal tunnel syndrome is defined by a quick development of median neuropathy induced by abrupt increase in carpal tunnel pressures, resulting in median nerve ischemia, and necessitating immediate decompression treatment [3, 4]. It is less common than chronic CTS and is frequently associated with a radius fracture, burns, and local infection/injections [3]. However, chronic CTS is more common in practice that lasts the persistent symptoms for months to years usually accompanied with the local, regional, and systemic causes [3, 5]. The most common aetiology of CTS is idiopathic; however, other probable causes may be classified into the local (tenosynovitis, hypertrophic synovium etc.), trauma including Colles fracture, dislocation of the carpal bone/s, recent/malunited fracture adjacent to the wrist joint, anatomical anomalies, tumours, regional, and systemic factors. Occupational factors such as repetitive stress injury to the flexor tendons of hand, for example, in computer professionals, data entry clerks, typists, pianists, guitarists, sitarists, and fine art painters may predispose to the development of CTS [3, 5–7].

Probable pathologies include such as an increase of pressure in the carpal tunnel contributed by carpal bones, stiffness of the synovium and flexor retinaculum, flexor tendon thickening and tightening during activity leading to restricted mobility of median nerve, deformation of median nerve [6–8], and venous congestion and arterial obstruction [9]. Nerve impairments of 5–10% cause disruption to intraneural blood flow, axonal transport, and nerve conduction [8, 10].

Several conservative treatments have been suggested for mild to moderate CTS, such as patient education, physical therapy agents, night splints/orthosis, local corticosteroid injections, nonsteroidal anti-inflammatory drugs, oral steroids, exercises, yoga, ergonomic modifications, and manual therapies with wide variations in the long-term effectiveness of these treatments [10–14]. Surgical carpal tunnel release has generally been successful [3, 11, 15]. The neurodynamic technique (NT) is effective in improving the excursion of the nerve, thereby improving the transmission of nerve impulses [16–19]. Several studies have supported the use of tendon gliding exercises (TGE) to break soft tissue adhesions and resolve symptoms in CTS [6, 20]. Applying tendon gliding exercises leads to stretching of the soft tissue adhesions in the carpal tunnel, thus improving the relative excursion of median nerve [6]. In addition, the efficacy of NT and carpal bone mobilization technique (CBMT) in participants with CTS showed more significant improvement in relieving the symptoms in their treatment group than control group, however showed equal effect when compared with each other [21–23].

Previously, many studies reported managing chronic CTS conditions by applying either NT or CBMT, etc., alone or combined with conventional interventions, including moist heat and electrotherapy, which could not target markedly to break soft tissue adhesions [6, 16–21]. Probably, it was a genuine and vital missing link/reason for not getting significantly different results in the trials. This study precisely utilized tendon gliding exercises to address the soft tissue adhesion for facilitating the action of NT and CBMT and intending to find out which one of the two manoeuvres (NT or CBMT) takes advantage of the effect of TGE and becomes more effective in the treatment of CTS. This study will help the clinicians/physiotherapists in finding the best possible treatment combination approach while managing the symptoms of chronic CTS.

## 2. Materials and Methods

### 2.1. Study Design

The study was based on two-arm parallel-group randomized comparative design.

### 2.2. Ethical Consideration

This study followed and abided by the ethical guidelines for the conduction of the research on human participants and obtained an ethical clearance from the research ethics subcommittee at King Saud University. The study was conducted in accordance with the guidelines on human research of the World Medical Association Declaration of Helsinki. All the participants returned a filled consent form for this study.

### 2.3. Sample Size and Sampling

The sample size was obtained using the effect size (1.36) from a previous similar study [16], a 0.05 significance level, and a power of 0.80; the required sample size was 20 (10/group). Considering a dropout rate of 30%, an adequate sample of 26 was determined [16]. A sample of 30 participants (24 females and 6 males) with a diagnosed case of unilateral chronic CTS were recruited from the outpatient department, physiotherapy department of the university hospital. A simple random sampling method of randomization (sequential grouping with even and odd numbers) was used in allocating the participants to the study groups.

### 2.4. Participants

Participants with only unilateral chronic CTS were screened and recruited for this study based on the inclusion and exclusion criteria. Inclusion criteria included criteria such as age between 30 and 59 years; both genders (males and females); pain, tingling, or paraesthesia in the hand including the thumb, index finger, middle finger, and the radial half of the ring finger for at least 6 to 9 months; pain intensity: VAS score (at the time of maximum pain over the past 24 hours) ranging between 4 and 7 cm on a 10 cm VAS scale; presence of at least two of the following physical signs; positive Tinel's sign (sensitivity-0.97; specificity-0.91) and positive Phalen's test (sensitivity-0.92; specificity-0.88); sleep disturbance caused by hand pain; and a positive nerve conduction study (NCS) test for the median nerve (distal motor latency (DML) >4.4 ms, difference between DML of median and ulnar nerves >1.1 ms). Participants with bilateral CTS and/or unilateral CTS who received any conservative treatment in the past 1 month; prior neurovascular surgery related to neck or upper limb in the past 6 months, neurovascular pathologic conditions of the neck or upper limb other than CTS, and systemic pathologies related to CTS, such as hypothyroidism, diabetes mellitus and RA; known tumour, mass, deformity in the hand or wrist; current pregnancy and chronic pain conditions such as fibromyalgia and myofascial pain syndrome including pain referred to the hand; CTS as the result of an upper extremity fracture or trauma to median nerve; prior surgery for CTS; severe thenar muscle atrophy; and known psychosocial problems were excluded from this study.

### 2.5. Procedure

**Figure 1:**
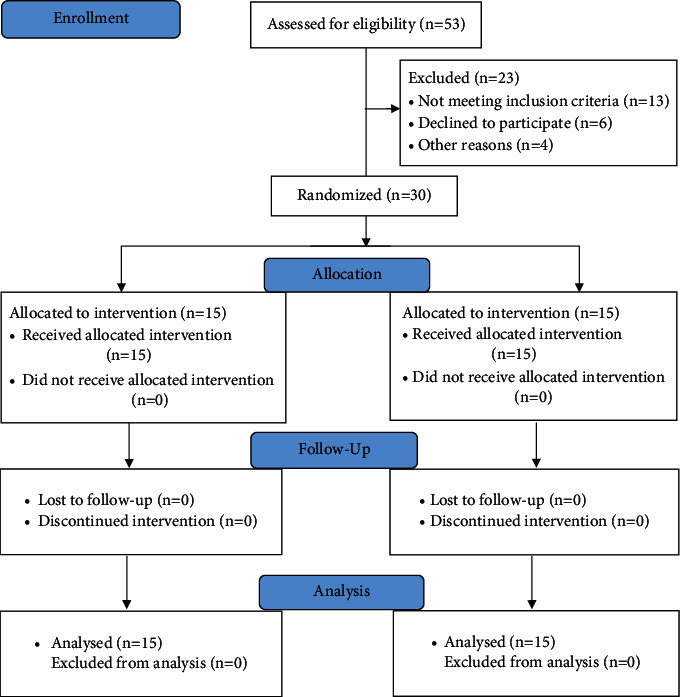
A CONSORT (2010) flow diagram including participants' enrolment, allocation, follow-up, and analysis of data.

All the participants were a diagnosed case of unilateral chronic CTS (a total of 30 hands) by orthopaedic surgeon and referred to the outpatient department physiotherapy of our university hospital. This study period extended to eleven months to be completed. All the participants were asked to fill a consent form and return to us prior to the commencement of the study. They were screened, recruited, and randomly allocated into both NT group and CBMT group. A simple random sampling method of randomization, using the sequential grouping with even and odd numbers, was used in allocating the participants to the study groups. The procedure included a sequential assignment of each participant into each group based on the odd and even numbers. So, the participants who picked up even numbers were assigned to the NT group, while those who picked up odd numbers were assigned to the CBMT group. The head physiotherapist performed the randomization procedure for allocating the participants to their groups. Both groups received their postulated interventions along with the full explanation of study protocol, procedure, assessment, evaluation, and precautions. Two specialist physiotherapists who were experts in either of the techniques delivered the NT and CBMT accordingly. One of them delivered the neurodynamic technique to NT group and another delivered the carpal bone mobilization technique to CBMT group. The intervention was delivered for a duration of 3 alternate days per week for 3 weeks to both the groups. The primary outcome measures including visual analogue scale (VAS), Boston Carpal Tunnel Questionnaire (BCTQ), hand-held dynamometer (HHD), and electromyograph (EMG) were used to assess the pain intensity, symptom severity and functional status, grip strength, and motor nerve conduction study, respectively. Two assessors (assistant physiotherapist and EMG lab technician) who were also kept blind to the study recorded the scores of all outcomes for all the participants at baseline (preintervention), 3 weeks postintervention, and follow-up 4 weeks postintervention. The study procedures (allocation, randomization, follow-up, and analysis) were explained using a consort (2010) flow diagram as shown in Figure 1.

### 2.6. Interventions

The NT group received a neurodynamic technique (median nerve mobilization) in addition to tendon gliding exercises while CBMT group received carpal bone mobilization in addition to tendon gliding exercises. The NT and CBMT were performed as a primary intervention; however, TGE was performed as an adjunct to the NT and CBMT.

#### 2.6.1. Neurodynamic Technique

**Figure 2:**
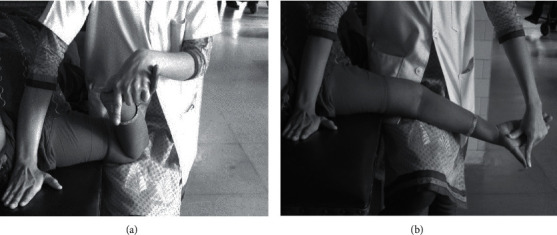
NT for median nerve ((a) elbow flexion with wrist extension; (b) elbow extension with wrist flexion).

This manoeuvre intends to yield an unrestricted gliding movement of the median nerve against the surrounding soft tissues inside the carpal tunnel. The joints were moved in such a way that stretched the nerve proximally while releasing it distally followed by a reverse combination. For the right side, procedure is as follows:

The participant laid supine on a plinth. The therapist stood in stride standing (right leg in front of the left one) on the right-hand side of the plinth, facing the participant. The participant's arm rested on the therapist's right thigh. The therapist's left hand held the participant's right hand. The participant's shoulder girdle was depressed by the therapist by pushing the right hand vertically down the plinth. The participant's shoulder was then taken into abduction (90°) and lateral rotation, the forearm was supinated and wrist, thumb, and fingers were extended. In this position, concurrent elbow flexion and wrist extension (Figure 2(a)) was alternated dynamically with concurrent elbow extension and wrist flexion (Figure 2(b)). The therapist alternated the combination of movements depending on tissue resistance. Speed and amplitude of movement was adjusted so that it produced no pain. The NT was performed in 2 sets of 5 minutes each with 1-minute rest between sets. It was performed three times per week for three weeks consecutively [16].

#### 2.6.2. Carpal Bone Mobilization Technique

**Figure 3:**
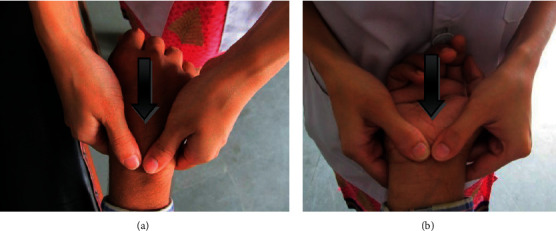
CBMT ((a) P-A glides; (b) A-P glides over carpal bones).

CBMT is the movement of the individual carpal bones in a posteroanterior (P-A) and an anteroposterior (A-P) direction in relation to the adjacent carpal bone, radius, ulna, or adjacent metacarpal. Participant was positioned in supine lying in the middle of the couch with the forearm resting on the couch either pronated or supinated. The specialist physical therapist stood by the participant's involved side beyond the hand, facing the participant's head. For P-A glide, the therapist positioned his hand to localize the forces on the carpal bone in such a way that the maximum breadth of the thumb tips was placed adjacent to each other on the appropriate carpal bone or intercarpal joint; the fingers were spread over the adjacent area of the hand for stability; and the arms and thumbs were positioned in a P-A direction as shown in Figure 3(a). For A-P glide, the thumbs contacted the palmar surface of the participant's supinated hand against the appropriate carpal bone or intercarpal joint; the fingers were spread over adjacent areas of the hand for stability; and the thumbs and arms were positioned in an A-P direction as shown in Figure 3(b). The P-A or A-P movement was produced by pressure from the therapist's arms being transmitted through the spring-like action of the thumbs against the appropriate carpal bone or intercarpal joint. The CBMT was performed in 3 sets with 30 repetitions in each set, keeping a gap of one minute between the sets. It was performed three times per week for three weeks consecutively [21].

#### 2.6.3. Supervised Tendon Gliding Exercises

**Figure 4:**
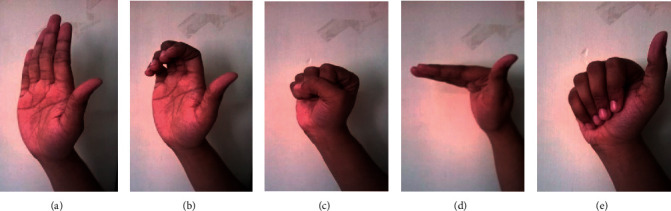
A supervised TGE ((a) straight; (b) hook; (c) fist; (d) table top; (e) straight fist).

In both the groups, tendon gliding exercises was carried out by the participant after applying the manoeuvre (NT or CBMT). The participant was in sitting position on a chair. The TGE involved sliding the flexor tendons of the hand by moving the fingers through the following five discrete positions: straight, hook, fist, table top, and straight fist positions as shown in Figures 4(a)–4(e). The TGE was actively performed by the participants who maintained each position for 7 seconds and repeated five times in each set for 3 sets, keeping one-minute rest between sets. It was performed three times per week for three weeks consecutively [24].

### 2.7. Outcome Measures

Pain intensity, symptom severity and functional status, grip strength, and motor nerve conduction study were assessed by visual analogue scale (VAS) [25, 26], Boston Carpal Tunnel Questionnaire [27, 28], hand-held dynamometer [28], and electromyograph [17].

#### 2.7.1. Pain Intensity

A visual analogue scale (VAS) was used to assess the intensity of pain. It is a long, 10 cm horizontal line marked with no pain at one end (0) and maximum unbearable pain at another end [10]. The participant was instructed to mark the intensity of pain on the horizontal line, and the score was noted for the evaluation. The interclass correlation coefficient (ICC), standard error of measurements (SEM), and minimum detectable changes (MDC) for VAS were 0.97, 0.03, and 0.08, respectively [25, 26]. The minimally important clinical difference in VAS pain score was reported to be 1.7 cm. The readings were taken at baseline i.e., 1 week preintervention (VAS1), 3 weeks postintervention (VAS3), and follow-up at one week post end of the intervention (VAS4).

#### 2.7.2. Symptom Severity and Functional Status

A Boston Carpal Tunnel Questionnaire (BCTQ) was developed by Levine et al. [28]. It is a patient-reported outcome measures which is valid, reliable, responsive, and acceptable to assess the symptom severity and functional status of the participants with CTS [27, 28]. The participants were asked to read each item/question and tick carefully for the numbers within 1 to 5 that well describe the condition/problem when filling the questionnaire. The SSS and FSS consist of 11 items and 8 items, respectively. The scoring value for each item is ranged within one to five. The average score is obtained by dividing the total score by the total number of questions for all items. A higher score indicates the decrease in functional capacity. The readings were taken at baseline i.e., week 1, preintervention (BCTQ1), week 3, postintervention (BCTQ3), and follow-up at one-week post end of the intervention (BCTQ4).

#### 2.7.3. Grip Strength

A Jamar Hand Dynamometer was used for measuring grip strength which has evidence of good to excellent test-retest reproducibility (*r* ≥ 0.80) and excellent interrater reliability (*r* ≥ 0.97) [29]. The participant was seated comfortably on a chair with forearms, legs, and back supported while the wrist was kept just at end of the armrest of the chair. The arm was at the side of the body with adducted and neutrally rotated, elbow flexed at 90°, forearm, and wrist in neutral position. The handle of the dynamometer was kept by participant's hand so that the great thumb and four fingers were round either side of the handle while the base of the dynamometer rested on the palm of the observer's hand as to negate the effect of gravity on the peak strength. The participants were motivated to squeeze as much tightly as possible and stop the squeezing once the needle of dynamometer stopped raising further. Thus, position of needle in dynamometer was read and recorded. Two further measurements were taken in a similar manner with 30 seconds of rest between each trial, and the average of the three readings was considered [29, 30]. The readings were taken at baseline i.e., 1 week preintervention (GS1), 3 weeks postintervention (GS3), and follow-up at one week post end of the intervention (GS4).

#### 2.7.4. Distal Motor Latency (DML) of Median Nerve (a) and the Differences between DML of Median and Ulnar Nerve (b)

Nerve conduction study (NCS) assesses the state of median nerve which has high sensitivity (*r* = 0.93) and good specificity (*r* = 0.87) [31]. The NCS was carried out by an electromyograph using a bipolar surface electrode. All measurements were taken in the supine position. To reduce the potential of temperature variability, participants were allowed to acclimate to room temperature for 10–15 minutes before testing. Ambient temperature was maintained between 24°C and 26°C [10]. For median nerve conduction, the arm was placed at the side of the body with palm facing upward. The recording and reference electrodes were placed at closest to the motor point of abductor pollicis brevis and 3 cm distal at first metacarpophalangeal joint, respectively. A supramaximal stimulation was introduced at the wrist (3 cm proximal to the distal wrist crease, region over the median nerve). For ulnar nerve conduction, the elbow was flexed to 135° wrist neutral. The recording and reference electrode were placed closest to the motor point of Abductor digiti minimi and on the proximal phalanx of the fifth digit, respectively. A supramaximal stimulation was introduced at the wrist (3 cm proximal to the distal wrist crease, region over the ulnar nerve) [29, 30]. The score for median nerve distal motor latency was recorded at baseline i.e., 1 week preintervention (NCSa1), 3 weeks postintervention (NCSa3), and follow-up 4 weeks postintervention (NCSa4). Similarly, the value for the difference between distal motor latency of median and ulnar nerves (NCSb) was recorded at baseline i.e., 1 week preintervention (NCSb1), 3 weeks postintervention (NCSb3), and follow-up at one week post end of the intervention (NCSb4).

### 2.8. Statistical Analysis

A software Statistical Package for the Social Sciences (SPSS Inc.v.19) was used for the analysis of the collected data. Descriptive statistics was used to summarize the participants' demographic details. All the outcome variables including VAS, BCTQ, GS, NCSa, and NCSb were interval/ratio level data. To determine the normality in the distribution of data, Shapiro–Wilk test was used. Paired and unpaired *t*-test were used to analyse the differences for the scores of the outcomes within and between the groups, respectively. The tests were applied at 95% confidence interval on *α* value set at 0.05, and results of the study were considered to be significant if *p* < 0.05.

## 3. Results

A total of 53 participants (female = 35; male = 12) were assessed for the eligibility in the study. 23 participants (female = 11; male = 6) were excluded from the study. Out of 23 participants, 13 did not meet the inclusion criteria, 6 declined to participate due to time constrain, and however, 4 denied to participate without any reason. A total of 30 participants (female = 24; male = 6) were randomly assigned into the NT group (*n* = 15) and CBMT group (*n* = 15). On average, women (73.33%) enrolled more than men (26.66%) in the study, the participants were of older age group (Mean age = 50.99). The participants' demographics data and all the outcome scores were distributed homogeneously and well matched (95% CI, *p* > 0.05) at the baseline. The mean scores for the age, male and female distribution in each group, and sides of the CTS involvement are presented in Table 1.

Each group showed a significant difference (95% CI, *p* < 0.05) for the scores of the outcomes VAS, BCTQ, GS, NCSa, and NCSb when compared to their baseline scores with 3 weeks postintervention and follow-up 4 weeks postintervention scores as presented in Table 2.

Similarly, comparison between the groups for the scores of the outcomes BCTQ, NCSa, and NCSb revealed a significant difference (95% CI, *p* < 0.05) at 3 weeks and follow-up one week post end of the intervention except for the scores of outcomes VAS and GS (95% CI, *p* > 0.05) as presented in Table 3.

In addition, compared to the magnitude of differences within each group at different time intervals, the *t*-value indicated the superiority of the NT group over the CBMT group for the outcomes BCTQ, NCSa, and NCSb and of the CBMT group over the NT group for the outcomes VAS and GS, as presented in Table 2.

## 4. Discussion

Most of the participants with CTS urge medical intervention to get relief from the symptoms and removal of their functional limitations [31]. Several conservative interventions have been used to treat CTS for the decades [12]. This study attempts to compare the efficacy of two manoeuvres i.e., NT and CBMT with TGE as a common adjunct. This study's findings showed a significant improvement within each group. The NT group achieved more remarkable improvement than the CBMT group regarding symptoms severity, functional status, and nerve conduction speed except for the pain and grip strength (*p* > 0.05).

Different types of nerve gliding exercises (sliding or tensioning technique) have been observed to be mechanically effective on the peripheral nervous system. The simultaneous movement or the position of an adjacent joint strongly influences the longitudinal excursion and strain on nerve. Neural tensioning technique is pretended to increase a relatively larger strain on the nerve; however, it seems to be contradicted in some conditions. Moreover, a sliding technique is considered to be less aggressive and more appropriate for the conditions like acute injuries (bleeding and inflammation), nerve irritation/entrapment, and postoperative management [32]. In this study, sliding technique was chosen that prevented overstretching and irritation of an already irritated and compressed median nerve in the carpal tunnel, which could have led to adverse effects.

From the results obtained after data analysis, it can be concluded that these manoeuvres (NT and CBMT) have positive results on participants with CTS with regards to pain. Following the interventions, 69% reduction in the intensity of pain was found in NT group and 66% that in CBMT group posttreatment in within-group analysis; however, no significant difference in pain intensity was noted in between-group analysis at 3 weeks postintervention. The results were also found to sustain in the follow-up period (4 weeks) in both the groups.

The results are in agreement with a previous study that investigated the effects of NT and CBMT in the treatment of participants experiencing CTS and found both treatment methods to be equally effective in reducing pain [21]. Further, it revealed that neural mobilization technique may minimize the pressure inside the nerve that leads to increase in intraneural and perineural blood flow [21]. Besides that, mobilization of carpal bones may influence the pressure in the nervous system which further helps in the dispersion of the existing oedema. The carpal tunnel including the flexor retinaculum is a part of the interface of the median nerve, and mobilization of the interface could therefore have an effect on any extra-neural component which is the cause of the problem. An intervention to the interface may be helpful in stabilizing the pressure gradients in the carpal tunnel and normalize the blood supply accordingly.

Median nerve mobilization reduces the scar tissues and mechano-sensitivity, and enhances the mechanical adaptability, of the nervous system which decreases the pain directly or indirectly, and allows less restricted movement of the body, as stated by Butler [17]. The soft tissue mobilization and nerve slider neurodynamic technique were delivered for a single session and found to be effective significantly in reducing pain even at one week postintervention [16]. Another study reported that the manual therapies act over the central pain control mechanism by activating the descending inhibitory pathway through the periaqueductal grey area to alleviate the pain and by mediating pain-related neuroplastic changes. Thus, the reduced intraneural pressure inside the carpal tunnel substantially alleviates the symptoms of neural hypoxia and nerve pain syndrome and desensitizes the nervous system through the peripheral stimulus (peripheral effect) [16]. However, one study reported that the effect of neural mobilization techniques is immediate rather than sustained like in *C*-fibre mediated thermal hypoalgesia among healthy adults [33]. Another study reported that the manual therapies including manipulation, mobilization, message, and neurodynamic techniques exert their effect by reducing the excitability of dorsal horn cells through the inhibition of temporal summation. Whereas, temporal summation is mediated by the *C*-fibre which advances the acute pain into chronic pain and maintain the chronicity of pain [34].

The within-group improvement in pain intensity in the NT group can be explained by the decreased mechano-sensitivity of the nervous system, reduced scar tissues in the carpal tunnel, the activation of descending pain inhibiting mechanism, neuroplastic changes associated with pain resulting in hypoalgesia, *C*-fibre mediated hypoalgesia, and reduced pressure in carpal tunnel leading to improved blood flow to the nerve resulting in the regeneration and healing. The positive effect in CBMT group might be due to reduced pressure in the tunnel relieving median nerve compression, dispersion of any existing intraneural oedema, and improved blood flow in the perineurium and epineurium which aids to heal the nerve and relieve ischaemic pain. The application of TGE could be responsible for the sustained effect of reduced pain in the follow-up period. By providing maximal differential gliding for both the flexor tendons of the hand, TGE helps to remove adhesion in the tunnel and enables longitudinal excursion of the median nerve. It exerts pressure on the median nerve without overstretching it, which may otherwise cause symptoms and discomfort [20].

The within-group analysis of nerve conduction study in both the groups demonstrated statistically significant improvement posttreatment and at follow-up with CBMT group showing better result compared to the NT group. These results have been supported by previous studies using nerve conduction studies in the treatment of CTS [18, 35]. One study stated that the symptoms like numbness, tingling, acute pain, with loss of nerve conduction are experienced in CTS due to microvascular insufficiency i.e., lessening of micro-nutrients and oxygen supply to the nerve which causes the nerve to lose its impulse conduction capacity slowly and formation of scars and fibrous tissues within the nerve [4]. The rationale in treating participants with neurodynamic technique and carpal bone mobilization is an attempt to improve the nerve conduction by reducing intraneural pressure through facilitating the axonal transport [21].

A previous study proposed that along with improving the axonal transport, NT accelerates the NCV by improving the flexibility of the structures around the joint along with contracted median nerve [17]. One study reported that the nerve mobilization of upper limb brought overall improvement in upper limb function by inhibiting the spasm and promoting the muscular tension in participants with traumatic brain injuries [35]. Similarly, declared in a study that median nerve mobilization in CTS participants reduced pressure in carpal tunnel leading to improved axonal flow, hence resulting in improved NCV [36]. Furthermore, few studies reported using ischaemic compression and massage therapy targeting the potential spots of entrapment of median nerve in the neck, arm, and forearm while treating the chronic CTS [12, 24]. A greater improvement in nerve conduction was seen posttreatment in the NT group compared to the CBMT group. A probable reason could be related to the release of adhesion and entrapment of the nerve anywhere along its entire course in the upper limb due to the application of neurodynamic technique compared to carpal bone mobilization in CBMT group which only addressed entrapment of the nerve at the site of carpal tunnel. The result of improved nerve conduction was retained in the follow-up in the groups. This could be probably due to healing of the damaged nerve along with optimization of the nerve function because of improved blood flow as the pressure was relieved by the application of neurodynamic technique and carpal bone mobilization [18]. Tendon gliding exercises enhanced differential gliding of median nerve relative to the nerve bed, thus preventing overstretching of the adhered nerve in the carpal tunnel [37].

Grip strength improved by 25.11% in the NT group and by 22.78% in the CBMT group following treatment, and the beneficial effect was found to sustain in the follow-up which was proved statistically. However, between-group analysis did not show statistically significant difference between the two treatment groups. The result of this trial is corroborated by a previous study that was conducted to find the immediate and long-term therapeutic effect of different combinations of interventions including tendon-nerve gliding techniques along with splinting, therapeutic ultrasound therapy along with splinting, and tendon-nerve gliding technique and therapeutic ultrasound therapy along with splinting on the grip and pinch strength in participants with CTS and found that all combinations of therapeutic interventions brought significant improvements in outcomes at immediate as well as at 8 weeks follow-up. However, the long-term participants' satisfaction questionnaire reported a more significant improvement in the symptoms among the participant's group who received the nerve-tendon gliding techniques and therapeutic ultrasound therapy along with splinting; and guided that the tendon-nerve gliding technique achieved more improvement in the excursion of the flexor tendons as well as brought the nonadherence between tendon sheaths (tenosynovium) and the structures around them in the carpal tunnel by remodelling and stretching them [37].

In a case report study, researcher revealed that the prime movers of thumb including the abductor pollicis brevis and opponens pollicis are needed to be fixed at the base of their attachment for pulling the thumb towards the other fingers while controlling the grasping function. A composite fixed base of attachment allows the prime movers to move the thumb freely. In contrast, free movable part of the thumb is required to be fixed, and the anchor of the thumb needed to be mobile while performing a sustained and powerful grasping or pinching function including holding a tool [9]. Thus, improving the mobility of the carpal bones by carpus mobilization might allow a better and powerful grip while it is measured with the hand-held dynamometer for grip strength measurement [34]. Several studies reported a negative relationship between pain and muscle strength suggesting that pain influences muscle activation as well as maximum voluntary muscle strength and reduction of pain is associated with increased maximum muscle strength and muscle activation [10, 38, 39]. Moreover, a correlation study advocated that higher motor NCV of median nerve was associated with increased grip and pinch strength as a result of enhanced motor nerve conduction [40]. The within-group improvement in the NT and CBMT groups in terms of grip strength could be attributed to improved excursion of flexor tendons, decreased pain, and enhanced median nerve motor conduction in both groups. In addition, increased mobility of the carpal bones contributed more to reducing pain and improving the grip strength in the CBMT group than the NT group.

Statistically significant improvement in function was found postintervention in within-group analysis in both the groups. Greater improvement was seen in the NT group (43.42%) as compared to the CBMT group (20.94%). This improvement was also found to sustain at one week follow-up. The result of this study was supported by a previous study [41], which declared that the neural mobilization combined with routine physiotherapy (rest, splint, transcutaneous electrical nerve stimulation, ultrasound therapy) was found to be effective in terms of function in the treatment group only. Further, suggested that it was a result of the adaptation of the hand with stretched state of the nerve which is usually needed in the performance of daily activities. In the previous studies, osteopathic manipulative treatment (OMT) including carpal bone mobilization along with other manoeuvres was found to be effective in improving the pain, range of motion, strength of flexors muscles, length of diameter of carpal tunnel cavity, flexibility of soft tissues, setting of restricted carpal bones, and decreasing the extra intraneural fluid in participants with chronic CTS [29, 42].

The improvement in function in the NT group and the CBMT group may be attributable to the effects due to reduction in inflammation or oedema and pain, in the carpal tunnel in both the groups [21, 41]. Other factors that affect the function of the hand are tingling, numbness, pain, paraesthesia, nerve conduction, mobility of nerve and tendon inside the carpal tunnel, and muscle strength [41].

A greater reduction in the BCTQ value in the NT Group suggests that adaptation mechanism associated with the participants treated by a neurodynamic technique where the affected limb was kept in a position where the median nerve gets stretched and that newly gained position might have had an adaptation effect which brought an improvement in hand function of the participants [41]. In addition, the effects of reduced pain, improved excursion of the nerve, enhanced nerve conduction, and improved grip strength could account for the within-group improvement in function during posttreatment and follow-up phases in both the NT and the CBMT groups.

Individual effectiveness of CBMT and NT to decrease pain and improve hand ROM and function, nerve conduction, and grip strength in patients with chronic CTS have been reported in previous studies [21–23]. It has also been reported that CBMT and NT are equally effective in decreasing pain and improving hand functions, nerve conduction velocity, and grip strength in CTS patients [21–23]. In addition, the TGE has been reported in removing adhesion, providing differential gliding of both flexor tendons and longitudinal excursion of the median nerve in the carpal tunnel [6, 20]. In this study, the magnitude of difference within groups revealed that the TGE added more advantages to the NT effects in improving hand functions and nerve conduction velocity by complete free longitudinal excursion and blood supply of the median nerve, removing adhesions in the carpal tunnel, and differential gliding the flexor tendon. However, the TGE added more benefits to the CBMT effects in reducing the pain and improving the grip strength by adding its effect to the flexor tendons and median nerve, reducing the tunnel pressure, washing out the excessive fluids, and providing free movements between the carpal bones. Therefore, the TGE might be considered a beneficial effect-enhancing adjunct to NT and CBMT in bringing about and maintaining the improvements in the chronic CTS's symptoms.

### 4.1. Limitations

The present study exhibited some limitations when it is viewed in light of duration of follow-up (short-duration) and use of a control group to establish the cause-and-effect relationship (absence of control group). A prospective study may be required to conduct a similar study in the future with a larger sample size to represent the major population by considering the effective sample size estimation, longer duration follow-up to determine the effects sustained over time, and include a control group to precipitate the actual effect of each intervention.

## 5. Conclusion

The study concluded that the neurodynamic technique was more beneficial than the carpal bone mobilization technique when incorporated with the tendon gliding exercises to enhance the nerve conduction velocity and functional status of the hand while managing the participants with chronic CTS. However, both NT and CBMT were equally effective in reducing the pain and improving the grip strength among CTS participants, also supported by a previous study [21]. In addition, the TGE contributed as a beneficial, effect-enhancing adjunct to the NT and CBMT. The physical therapists should apply either of the combinations of interventions according to their treatment goal while treating the participants with chronic CTS.

## Figures and Tables

**Table 1:** Participants demographics distribution between the groups (*n* = 15/group) using Shapiro–Wilk test (95% CI).

Variables		NT group (*n* = 15)	CBMT group (*n* = 15)	*p* value
Age (mean ± SD)		50.66 ± 3.49	51.33 ± 4.98	>0.05
Gender	Male	2	4	>0.05
	Female	13	11	>0.05

Side	Dominant	12	11	>0.05
	Nondominant	03	04	>0.05

Nonsignificant value if *p* > 0.05; SD: standard deviation; *n* = no. of participants in each group.

**Table 2:** Pairwise comparison for the scores of outcomes within groups using paired *t*-test.

Outcomes	Outcomes scores (mean ± SD)			W3-W1		W4-W3		W4-W1	
	Baseline	3 week	4 week	*t*	*p*	*t*	*p*	*t*	*p*
*NT group (n* *=* *15)*									
VAS	6.3 ± 0.65	1.95 ± 0.44	2.02 ± 0.49	22.62	0.001^*∗*^	−0.91	0.379	20.89	0.001^*∗*^
BCTQ	2.28 ± 0.32	1.29 ± 0.17	1.3 ± 0.15	14.26	0.001^*∗*^	−1.01	0.33	13.75	0.001^*∗*^
GS	17.08 ± 2.02	21.37 ± 3.28	21.26 ± 3.34	−10.12	0.001^*∗*^	0.87	0.398	−9.91	0.001^*∗*^
NCSa	5.54 ± 0.38	4.46 ± 0.43	4.48 ± 0.44	9.78	0.001^*∗*^	−1.94	0.073	9.41	0.001^*∗*^
NCSb	2.29 ± 0.38	1.45 ± 0.28	1.5 ± 0.28	8.68	0.001^*∗*^	−1.97	0.068	8.83	0.001^*∗*^

*CBMT group (n* *=* *15)*									
VAS	6.2 ± 0.47	2.12 ± 0.58	2.3 ± 0.58	25.22	0.001^*∗*^	−2.82	0.014^*∗*^	23.71	0.001^*∗*^
BCTQ	2.3 ± 0.47	1.85 ± 0.57	1.88 ± 0.56	6.27	0.001^*∗*^	−0.52	0.613	4.86	0.001^*∗*^
GS	17.42 ± 1.2	21.39 ± 2.07	21.04 ± 2.06	−13.17	0.001^*∗*^	3.56	0.003^*∗*^	−11.75	0.001^*∗*^
NCSa	5.51 ± 0.32	4.85 ± 0.44	4.97 ± 0.49	7.67	0.001^*∗*^	−1.70	0.11	5.62	0.001^*∗*^
NCSb	2.33 ± 0.40	1.98 ± 0.43	2.0 ± 0.45	8.27	0.001^*∗*^	−0.76	0.461	3.98	0.001^*∗*^

W1: preintervention scores at baseline; W3: postintervention scores at 3 weeks; W4: Follow-up scores at one week post end of the intervention; SD: standard deviation; t: *t*-test statistics; *p*: level of significance; ^*∗*^Significant value if *p* < 0.05; VAS: visual analogue scale; BCTQ: Boston Carpal Tunnel Questionnaire; GS: grip strength; NCSa: nerve conduction study for median nerve; NCSb: nerve conduction study difference between median and ulnar nerve.

**Table 3:** Between-group comparison for the scores of the outcome measures using unpaired *t*-test.

Outcomes	NT group vs. CBMT group (*n* = 15/group)								
	Baseline			Week 3 postintervention			Week 4 (follow-up)		
	∆MD±∆SD	*t*-value	*p* value	∆MD±∆SD	*t*-value	*p* value	∆MD±∆SD	*t*-value	*p* value
VAS	0.10 ± 0.18	0.16	0.87	−0.17 ± 0.14	0.87	0.389	−0.28 ± −0.09	1.41	0.16
BCTQ	−0.02 ± −0.15	0.37	0.70	−0.56 ± −0.4	3.63	0.001^*∗*^	−0.58 ± −0.41	3.80	0.001^*∗*^
GS	−0.34 ± 0.82	0.54	0.58	−0.02 ± 1.21	0.02	0.98	0.22 ± 1.28	0.21	0.82
NCSa	0.03 ± 0.06	0.26	0.79	−0.39 ± −0.01	2.45	0.02^*∗*^	−0.49 ± −0.05	2.86	0.008^*∗*^
NCSb	−0.04 ± −0.02	0.25	0.79	−0.53 ± −0.15	3.94	0.001^*∗*^	−0.50 ± −0.17	3.90	0.001^*∗*^

*t*-value: *t*-test statistics; *p* value: level of significance; ^*∗*^Significant value if *p* < 0.05; ∆MD: mean differences; ∆SD: standard deviation difference; VAS: visual analogue scale; BCTQ: Boston Carpal Tunnel Questionnaire; GS: grip strength; NCSa: nerve conduction study for median nerve; NCSb: nerve conduction study difference between median and ulnar nerve.

## Data Availability

The datasets used and/or analysed during the current study are available from the corresponding author on reasonable request.

## Abbreviations

CTS: Carpal tunnel syndrome
NT: Neurodynamic technique
CBMT: Carpal bone mobilization technique
TGE: Tendon gliding exercises
VAS: Visual analogue scale
BCTQ: Boston Carpal Tunnel Questionnaire
GS: Grip strength
NCS: Nerve conduction study
NCSa: Motor latency of Median nerve
NCSb: Motor latency difference between median and ulnar nerve.
